# Managerial Networking and Frugal Innovation: Situational Leadership Perspective

**DOI:** 10.3389/fpsyg.2022.948530

**Published:** 2022-07-05

**Authors:** Wei Xuecheng, Qaisar Iqbal

**Affiliations:** ^1^School of Management, University Sains Malaysia, George Town, Malaysia; ^2^School of Economics and Management, Inner Mongolia Normal University, Hohhot, China; ^3^Centre for China-India-Pakistan Studies, Sichuan University of Science and Engineering, Zigong, China

**Keywords:** networking, sustainability, leadership, resource-constraint, SMEs, frugality

## Abstract

This study aimed to examine the integrated relationships of business ties, political ties, sustainable leadership, and frugal innovation. The correlation was assessed with the consideration of social network and situational leadership theories. Data was collected from 363 Small and Medium Enterprises (SMEs) employees in Pakistan with a response rate of 72.60%. Accordingly, the Partial Least Squares-Structural Equation Modeling (PLS-SEM) was employed to examine the validity of the proposed hypotheses. Empirical findings confirmed the significant impact of managerial and business ties on frugal innovation, strengthened by sustainable leadership. However, data analysis negated the positive impact of political ties on frugal innovation, which remains non-significant with the introduction of sustainable leadership among Pakistani SMEs. Hence, future studies are recommended to explore other conditional factors and mediating mechanisms in the relationship between managerial ties and frugal innovation. This idea can bring a deeper insight into the vital role of networking. To the best of the author’s knowledge, no prior study is available about the integrated relationships of managerial ties, frugal innovation, and sustainable leadership. This study enriches the literature in innovation management, especially concerning the social network and situational leadership model.

## Introduction

Sustainable development goals (SDGs) are deemed an authentic means to cope with global challenges of social inequalities, environmental damages, and poverty (United Nations., 2015; Halisçelik and Soytas, 2019). The goals focus on bridging the gap between intra- and inter-nation inequality and adopting a triple bottom line approach to integrate sustainable performance’s economic, social, and ecological dimensions (Agbedahin, 2019). In essence, individuals and organizations can satisfy their needs without adversely affecting the ability of future generations by adopting a sustainable development approach (World Commission on Environment and Development., 1987).

Frugal innovation has emerged as a powerful strategy to accomplish SDGs (Brem et al., 2017) because of its focus on redesigning products and services for low to middle-income consumers (Knorringa et al., 2016; Rosca et al., 2018; Iqbal et al., 2021). Society is encouraged to reduce natural and financial resource consumption (Herstatt and Tiwari, 2020). This form of innovation attempts to use minimum resources of various types during different stages of a product’s life cycle. However, limited empirical evidence is available about the antecedents and outcomes of frugal innovation despite being a novel research area (Rosca et al., 2018; Iqbal et al., 2020; Hossain et al., 2021).

Previous studies have empirically confirmed various elements that significantly spur frugal innovation, i.e., internal capabilities, surrounding innovation ecosystems, and overarching institutional frameworks (Fischer et al., 2021). Others include firm-level resource constraints (Ploeg et al., 2021), internal and external sources of knowledge (Dost et al., 2019), and bricolage capability (dos Santos et al., 2020). Furthermore, transformational and sustainable leadership (Iqbal et al., 2020b; Lei et al., 2021) exhibited significant frugal innovation predictors. Frugal innovation adoption is considered feasible from the perspective of sustainable development. However, compared to large firms, Small and Medium Enterprises (SMEs) face challenges in coping with contradictory knowledge processes and dysfunctional competition, especially in developing countries.

The above phenomenon is due to the SMEs’ lack of sufficient resources, Research & Development (R&D) investments and hierarchical administrative systems (Ahmad et al., 2017; Cai et al., 2019). This predicament impedes their progress in effective innovation strategy. Hence, managerial ties play a crucial role in applying innovation strategies based on social networking theory. This approach is achieved by providing necessary resources, market information and securing limited government resources (Petruzzelli, 2011; Zeng et al., 2014; Wu and Peng, 2020).

Managerial ties are classified into business and political ties, fostering innovation (Gao et al., 2008) and are strongly related to business model innovation (Wang et al., 2017). The concept is positively related to the inbound-open innovation but does not influence outbound open innovation (Naqshbandi and Kaur, 2014). A positive relationship of ties was reported with firms and government offices with the opportunity to capture (Li et al., 2014). Previous studies indicated inconsistent findings on the business ties-innovation relationship. For instance, business ties positively affect multiple innovation facets, including green (Zhang and Wang, 2022), process (Shu et al., 2012), and product (Wu, 2011; Shu et al., 2012; Sami et al., 2019). Others include radical (Shen et al., 2019), exploitative (Wu and Peng, 2020), exploratory (Su and Yang, 2018), and firm (process and product).

However, Wu and Peng (2020) reported a non-significant relationship between business ties and exploratory innovation. Business ties possess an inverted U-shape relationship with radical (Chen et al., 2014) and product innovation (Gao et al., 2017). Similarly, extant literature offers mixed findings regarding the political ties-innovation relationship. Political ties promote various innovation factors, i.e., radical (Chen et al., 2014) and exploratory innovation (Wu and Peng, 2020). Nevertheless, it does not affect product (Sami et al., 2019), exploitative (Wu and Peng, 2020), or exploratory innovations (Su and Yang, 2018). Accordingly, the concept exhibited an inverted U-shaped effect on green (Zhang and Wang, 2022) and product innovations (Wu, 2011). Furthermore, Gao et al. (2017) concluded with a U-shaped effect of political ties on product innovation. There is scarce empirical evidence regarding the correlation of business and political ties with frugal innovation. Therefore, this study extends the literature on frugal innovation by considering the social networking theory and empirically examining the role of managerial ties in frugal innovation.

In SMEs, managers substantially contribute to the firms’ innovation through their leadership (Su and Yang, 2018; Wu and Peng, 2020). They are a vital influence in strategic innovation compared to large manufacturing firms. This phenomenon is due to the perception of greater freedom, discretion, high responsibility, and their role on the operational and strategic sides (Cao et al., 2010; Mura et al., 2014). Based on the situational leadership theory (Hersey and Blanchard, 1969), no single leadership style fits every situation. Influential leaders adapt themselves to the requirements of different situations and the nature of the work required (Hersey and Blanchard, 1969). In other words, sustainable development entails sustainable leadership, which is viewed as highly effective (Kantabutra, 2017; Hallinger, 2020).

The focus on a shared vision will result in a system of valuing employees, capacity building, sustainable change, effective relationship management, socially responsible behavior, and long-lasting results (Avery and Bergsteiner, 2011). The leaders of SMEs could utilize social networking to help with strategic decision-making (Hyypiä and Khan, 2018). Previous studies evaluated the moderating impact of collaborative culture (Le, 2021), market and technological turbulence (Dost et al., 2019), and bricolage (Iqbal et al., 2021a) on the “sustainable leadership-frugal innovation” relationship. Thus, this study considers the research gap based on situational leadership theory by examining the impact of managerial ties on frugal innovation, especially among SMEs in sustainable leadership.

This effort contributes in three ways; firstly, the current study enriches the literature on inconsistent findings on the managerial ties-innovation relationship by providing evidence from a developing country. Moreover, no prior study is available on the integrated relationship of sustainable leadership, and frugal innovation followed by managerial, business, and political ties. Thus, the research gap is fulfilled by providing empirical evidence from SMEs in a developing country, namely Pakistan. Secondly, insights related to the managerial ties-frugal innovation relationship to the literature are enriched based on social networking theory. Thirdly, current research enriches the literature from the perspective of situational leadership theory.

Previous studies focused on the impact of sustainable leadership as an independent variable on business resilience (Avery and Bergsteiner, 2011), social innovation, and sustainable and environmental performance (Iqbal and Ahmad, 2020; Iqbal et al., 2020a,b, 2021). Others include psychological safety (Sulasmi et al., 2020), and creativity (Javed et al., 2021). In this study, the authors consider sustainable leadership as an environmental factor. Such leadership is further elaborated on how it affects managerial ties-frugal innovation relationships in SMEs vis-à-vis situational leadership theory.

This article is structured as follows. The coming section explains about theoretical background and hypotheses development. In the third section, the research methodology is written in detail. The fourth section offers the empirical findings and is followed by discussions. In this section, the authors also emphasize the research implications. In the end, there are limitations of the current study and directions for future research.

### Theoretical Background

In a social system, innovations flourish between individuals or organizations. The relationship design between individuals or organizations who initiate, communicate and adopt innovations is considered a social network (Liu et al., 2017). Such connections exist in the shape of friendship, communication, advice, or social support (Acquaah, 2012). The process to diffuse innovation is actually a networked process. Innovation always emerges because of inter-connected communication within a social network (Imran et al., 2018). According to social network theory, social relations transmit information, direct individual or organizational influence and drive attitudinal or behavioral change (Cao et al., 2010). According to social networking theory, organizations can secure scarce resources such as network resources and establish managerial ties (Li et al., 2020; Wu and Peng, 2020). The networking activities help organizations develop both business ties and political ties (Peng and Luo, 2000; Sheng et al., 2011). Such ties and boundary-spanning activities are sources of competitive advantage for organizations (Zhang et al., 2019). In developing economies, organizations access information and knowledge and cope with resource constraints through managerial ties (Peng and Luo, 2000; Li et al., 2008). Therefore, the present study examines the direct impact of managerial ties and their two dimensions- business ties and political ties on frugal innovation.

From the perspective of sustainable development goals, frugal innovation offers value-added, cheap and easy-to-use products balancing social and organizational needs along with environmental challenges (Niroumand et al., 2021). According to situational leadership theory, every situation is unique and requires a specific type of leadership (Vecchio, 1987). Sustainable leadership has emerged as the most effective leadership style for dealing with sustainability challenges (Avery and Bergsteiner, 2011; Gerard et al., 2017; Iqbal and Piwowar-Sulej, 2022). Under the umbrella of situational leadership theory, sustainable leaders promote sustainability at the organizational level (Hallinger, 2020). Therefore, the current research portrays the conditional effect of sustainable leadership on the relationship of managerial ties and its two dimensions with frugal innovation.

### Hypothesis Development

#### Managerial Ties and Frugal Innovation

Managerial actions are embedded in networks of interpersonal relations (Abosag and Naudé, 2014). The social networking theory suggests that firms can secure information and resources through networking, facilitating the application of differentiation strategy (Park and Luo, 2001). As a differentiation strategy, frugal innovation requires the availability of complementary and regulatory resources (Cao et al., 2009; Li et al., 2013; Zhao et al., 2016). In essence, managerial ties enable firms to access external sources (Li et al., 2008), stimulating innovation. The concept concerns the boundary-spanning activities of managers and their associated interactions with external parties (Thongsri and Chang, 2019).

Managerial ties are divided into two categories, namely business and political ties (Li et al., 2008, 2011; Sheng et al., 2011), where the former refers to the relationship with buyers, suppliers, competitors, and other stakeholders (Kull et al., 2016). Meanwhile, the latter indicates the relationship with political leaders, industrial bureaus, regulators, and supporting organizations within the government (Peng and Luo, 2000). The two play a crucial role in acquiring external resources, which are different in nature (Fan et al., 2012; Zhang et al., 2019).

There is always a dire need to reduce uncertainties and mitigate risks while innovating. Hence, business ties help firms obtain scarce resources and market intelligence, mitigate uncertainties, and gain legitimacy *via* the network members (Chung et al., 2016).

High product creativity requires positive working relationships with diverse stakeholders (7, 56, 78). Firms can apply creative approaches to solve new problems under frugal innovation. Business ties are concerned with the firm’s relationship with customers, suppliers, competitors, and other stakeholders in a geographical area, stimulating innovative activities (Boons and Lüdeke-Freund, 2013). Frugal innovation requires firms to understand customers’ emerging needs, market inefficiencies, ingenious application of resources, and adopt a holistic rethinking approach. Close relationships with customers facilitate firms to understand genuine market needs and apply effective differentiation strategies (Sheng et al., 2011; Mashahadi et al., 2016).

Nevertheless, firms face challenges in accessing codified information from public sources. They can enjoy reliable market information, quality materials, on-time delivery, and good services through business ties with suppliers (He et al., 2018). The speed at which information is acquired and disseminated to managers *via* firms’ business ties is faster than the acquisition *via* formal channels (Sheng et al., 2011). Thus, close relationships with suppliers help firms to squeeze costs.

A close association with competitors can initiate firms to conduct information exchange and inter-firm collaboration. This idea will ultimately reduce implicit collusion (Li et al., 2014), a critical factor in frugal innovation (Le, 2021). Furthermore, communication with managers promote sharing of technology and knowledge (Li et al., 2008; Wang and Chung, 2013), fostering collaboration with different stakeholders (Wu and Peng, 2020). Similarly, ties with universities and research institutes spur innovation by making knowledge or resources accessible to firms *via* collaboration.

Political ties concern the link between government institutes and officials (Chung et al., 2016). Government officials have discretionary power to allocate strategic resources and approve projects (Peng and Luo, 2000). The development of frugal innovation highly relies on generic and regulatory resources. Accordingly, these officials bolster innovation activities by facilitating knowledge diffusion, funding, technology transfer and project management (Hofman and de Bruijn, 2010). However, regulatory resources have no direct impact on frugal innovation but substantially influence its capacity and scale of production (Zhang et al., 2019).

Government institutes facilitate innovation activities through learning opportunities (Cardoza et al., 2016), enabling firms to adopt differentiation strategies. Moreover, the government is interested in investing in innovation to enhance human and creative capital (Ye and Nurse, 2013). For instance, they conduct skill development programmes, promote entrepreneurship, and provide access to funding/financing (Boccella and Salerno, 2016; García et al., 2018). In developing countries, solid political ties ensure the availability of regulatory resources such as bank loans, tax exemptions, land, and legal protection, followed by industrial and policy information (Li et al., 2011; Sheng et al., 2011; Zhao et al., 2016). Close relations with government officials facilitate the acquisition of leapfrog technology, human capital and institutional support against competitors, leading to positive firm outcomes (Luo and Park, 2001).

Strong political ties ensure government officials’ assistance in adverse situations (Sheng et al., 2011). Furthermore, this idea offers opportunities for firms to collaborate and seek innovative solutions to challenges within their industries (de Zubielqui et al., 2015). Moreover, firms can identify unattended market inefficiencies and explore unique applications to reduce costs. Hence, public offices should establish training centers to promote knowledge sharing among stakeholders, facilitating collaboration.

Managerial ties enable firms to access external knowledge, technology, and resources, which in turn, improve existing systems and procedures (Cardoza et al., 2016). This idea reduces the firms’ cost and time in developing knowledge internally and minimizes risks (Papa et al., 2018). Therefore, practical exploration and internal integration of external ideas can be established. Ultimately, this concept helps them acquire knowledge and allocate resources from the external environment, utilized to spur frugal innovation.

H1: Managerial ties significantly influence frugal innovation.
H1a: Business Ties significantly influence frugal innovation.
H1b: Political ties significantly influence frugal innovation.

#### The Moderating Role of Sustainable Leadership

Organizational initiatives are highly dependent on (Koednok, 2011) the personal aspects of leadership, such as values and moral principles, a driving factor in sustainable initiatives (Renwick et al., 2013). Under the leadership style theory, a specific management approach is more effective for accomplishing organizational goals in certain situations (Wang et al., 2017). The leadership situation theory matches an administration style that aligns with the followers’ ability and enthusiasm in a specific situation (Hersey and Blanchard, 1969).

Sustainable leadership has emerged as the most effective approach to sustainability challenges such as climate change, biodiversity, cultural conflicts, and economic integration (Hallinger, 2020; Visser and Courtice, 2020; Iqbal et al., 2021). This concept focuses on the values, ethics, emotions, norms, and long-term social, economic, and environmental goals. Thus, this form of leadership encourages followers to perform beyond their expectations, developing stakeholders’ perceptions of the work environment (Wang et al., 2021). Leadership, knowledge sharing, and collaboration based initiatives are vital to fostering frugal innovation (Iqbal et al., 2021; Le, 2021).

The core objective of frugal innovation is to promote intergenerational equity and environmental justice (Von Zedtwitz et al., 2015), a crucial concern in sustainable leadership (Bulmer et al., 2021). Its practices revolve around practical communication skills and awareness of diverse stakeholders’ cultural backgrounds and values. Others include open communication, collaboration-based activities, updated information about current operations, knowledge sharing, trustworthiness, insight, and shared goal (Dalati et al., 2017). Sustainable leaders frequently ask questions about their operations, premises, and principles to identify opportunities and deal with problems. This idea ultimately spurs innovative culture (Osagie et al., 2016), enabling organizations to develop frugal products and services.

A conducive environment must be created to enrich resilience, strength, and vitality among its partners (Antunes and Franco, 2016). This idea can be achieved by enforcing effective relationship management, which is the foundation for FI and encouraging exchanging and integration of information with external partners (Hossain and Sarkar, 2021; Iqbal et al., 2021). In essence, organizations with excellent relationships with external parties are more likely to improve FI (Lu et al., 2020). A collegial relationship is crucial, especially with stakeholders, to understand their cultural differences and play the role of liaisons to foster consensus among parties (Lans et al., 2014; Armani et al., 2020).

Innovation is never free from risk; thus, a leader’s response and tolerance level to failure determine the followers’ work behavior (Alshebami, 2021). In sustainability, R&D must be promoted to facilitate individuals with tolerance and encouragement while seeking innovation in organizations (Iqbal et al., 2021). This move will reduce the pressure on stakeholders caused by uncertainty, improving their willingness to seek incremental and radical ideas (Akinola et al., 2019). Additionally, the participation of stakeholders can be enhanced, specifically in the work environment, positively affecting creativity (Sung et al., 2017).

From a political perspective, sustainable leaders are generally well versed in the ecological policies of their local government. These leaders can convince the government and its entities about the necessity of sustainability (Leal Filho et al., 2017) through training sessions. As a result, the availability of continuous funding can be ensured for stakeholders while creating solutions for all parties (Leal Filho et al., 2020). Henceforth, based on situational leadership theory, the following hypotheses are developed.

H2: The relationship between managerial ties and frugal innovation strengthens in the presence of high sustainable leadership.
H2a: Sustainable leadership significantly strengthens the relationship between business ties and frugal innovation.
H2b: Sustainable leadership significantly strengthens the relationship between political ties and frugal innovation.

**FIGURE 1:**
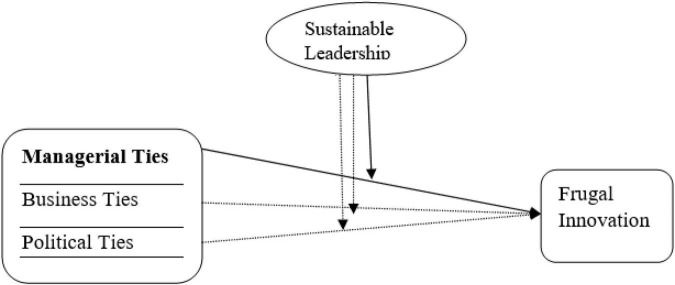
Research framework.

Referring to above propositions, current research framework is exhibited in below Figure 1.

### Research Methodology

#### Sample and Data Collection

Recent sustainable challenges have emerged, such as severe climate, poverty gap, and biodiversity, necessitating frugal innovation. This form of innovation is crucial in diverse health, manufacturing, agriculture, and automotive industries (Cunha et al., 2014; Hossain et al., 2021). Various SMEs create employment opportunities, contributing to export, though they are responsible for significant pollution of air and water pollution, followed by waste generation. Notably, SMEs substantially contribute to the economy of any country, though the implementation of the concept is still far from reality (Hyypiä and Khan, 2018). As part of the Frontier Asia Region (Iqbal et al., 2021a), Pakistani SMEs make up 30 per cent of the country’s GDP (Small and Medium Enterprises Development Authority, 2011). Thus, practitioners and academicians continuously raise their voices about the severe consequences of sustainable challenges to the Frontier Asia Region (Tonby et al., 2019; Jonathan Woetzel, Tonby et al., 2020).

By definition, SMEs in Pakistan are any type of firm comprising 250 employees (Small and Medium Enterprises Development Authority, 2011). The current research employed screening questions to guarantee the validity of participants. Hence, this study is conducted among SMEs in Pakistan, owing to the sample size, which plays a vital role in generating valid and reliable results. Accordingly, regarding the G*Power application (Faul et al., 2009), a minimum sample size equal to 85 participants must be met, ensuring the delivery of valid empirical findings.

Moreover, extant literature reported a 35.7 per cent average response rate in social research, where the standard deviation is ± 18.8 (Chen et al., 2018). This study assessed the assistance from personal ties in the SME sector, considering the time, financial, and low response rate in social studies. A convenience sampling approach was utilized by employing online survey forms sent *via* emails to 500 representatives of SMEs in Lahore, Faisalabad, Sialkot, Gujranwala, and Rawalpindi. In total, 370 questionnaires were collected after 3 months of distributing the survey to participants.

#### Measurement

In this study, the survey form included four categories: demographic information, independent variables, dependent variable, and moderator, comprising 27 items of continuous variables. The Likert-type scale is utilized to measure the items, a popular approach among academicians, albeit linked to acquiescence bias response (Iqbal et al., 2020c). Higher categories of Likert-point scales are deemed sources of cognitive burden and lower data quality (Robinson, 2018). Based on recommendations (Revilla et al., 2014), a five-point Likert scale was selected to collect data from SME employees in Pakistan, extending from strongly disagree (1) to strongly agree (5).

The authors ensured the accuracy of measurement scales by using the back-translation approach. Initially, we prepared a survey form in English, translated into Urdu (Native Language) by experts from the National University of Modern Languages, Islamabad. Finally, a professional translator service reverted the translation into English. After comparing the surveys in both Urdu and English, no semantic difference was found, thus ensuring both survey forms are identical. Additionally, the measurement items of all three continuous variables were presented in Appendix A. This study measured managerial ties as a second-order reflective-formative construct. Meanwhile, the first-order reflective constructs are business and political ties. Accordingly, four items were employed to measure the political ties and three items measuring business ties (Peng and Luo, 2000). The business ties construct entails the assess leaders’ relationships with customers, suppliers, and competitors.

Concurrently, leaders’ relationship with local political offices, tax authorities, public banks, and regulators comes under the umbrella of political ties. This scale was adopted by Wu and Peng (2020), who found it reliable. Cronbach’s alpha values of the business and political ties are 0.823 and 0.803, where sustainable leadership is the moderator. We adopted 15-items of sustainable leadership from McCann and Holt (2010) study. Sustainable leaders are highly concerned with developing effective relationship management, inspiring stakeholders, establishing a conducive working environment, and aligning the needs of stakeholders. In this study, the alpha value is 0.943, indicating high reliability. This result is in reference to a similar measurement scale from Iqbal and Piwowar-Sulej (2022) findings (alpha = 0.668).

This study has taken frugal innovation as a formative construct centered on customer orientation, low resource consumption, simple, mega-scale production, and value. The 05-items scale of frugal innovation, which is also a dependent variable, was adapted from the study of Iqbal et al. (2021). In organizations, leaders possess the discretionary power of decision-making. In other words, their personality attributes potentially affect organizational level outcomes (Lee et al., 2018). A crucial factor in selecting different strategic choices includes educational background (Shim and Okamuro, 2011).

Moreover, there are various predictors of different management experiences, namely age, gender, experience, and working years in a specific position. This idea drive leaders to take different decisions by perceiving the advantages and disadvantages of different types of innovation (Wu and Peng, 2020). Therefore, the current study has included gender, age, qualification, experience (in years), and position (in years) as control variables.

#### Data Analysis

This study appears complex due to its explanatory nature, encompassing the integration of reflective and formative constructs and investigating mediating effects. The reflective constructs include business ties and political ties, while the formative constructs include frugal innovation and managerial ties. In such scenarios, the partial least structural equation modeling (PLS-SEM) was selected for data analysis to investigate the indicator reliability, internal consistency reliability, and construct validity of reflective constructs. Notably, the results from this method are considered more valid than that of covariance-based structural equation modeling (CB-PLS) (Ringle et al., 2020).

The indicator reliability is acceptable provided its loading is more significant than 0.50; otherwise needs to be removed if its value lies below 0.40 (Chin, 1998). Furthermore, the internal consistency reliability was assessed based on Cronbach’s alpha and composite reliability (CR). For explanatory research, the values of CR and Cronbach’s alpha (α) higher than 0.70 indicate acceptable internal reliability of the measurement scales (Hair et al., 2020).

The convergent validity requires an average variance extracted (AVE) greater than 0.50. Subsequently, the Fornell-Larcker criterion, cross-loadings and Heterotrait-Monotrait (HTMT) ratios are conducted to assess the construct’s discriminant validity.

For discriminant validity, the square root of the AVE of each construct is required to be higher than its correlation with other constructs in the model. Meanwhile, the cross-loading criterion for the discriminant validity requires loading values of each construct indicator greater than all of its cross-loadings. There is acceptable discriminant validity provided the HTMT ratio is lower than 0.90 (Henseler et al., 2015). The present research included frugal innovation and managerial ties as formative constructs. However, each indicator or dimension of the formative construct indicates a different facet; thus, it is useless to assess their internal consistency or reliability (Petter et al., 2007).

The validity of the formative construct is evaluated at the indicator and construct level. The significance of the indicator weighs the presence of validity (Hair et al., 2020). Moreover, the variance inflation factor (VIF) was used to assess the validity of the formative construct, where its value must be less than 3.3 (Diamantopoulos and Siguaw, 2006). Meanwhile, the managerial ties are categorized under the second-order reflective-formative construct. The three approaches to assessing hierarchical order construct are repeated indicator, two-stage h, and hybrid (Becker et al., 2012). In this study, first-order constructs of business ties and political ties exhibit no equal number of items, and second-order construct-managerial ties are endogenous. Hence, regarding the recommendations of Duarte and Amaro (2018), a two-stage approach is applied to assess the validity of managerial ties.

## Results

### Data Screening

Each item in the online survey form has undergone mandatory checks, ensuring the absence of missing data. Accordingly, 363 valid questionnaires were left after removing 07 survey forms with invalid responses, revealing a response rate of 72.60%. The Z-score analysis was then conducted to assess the presence of univariate outliers. This study is free of univariate outliers as the values of all datasets were less than 3.3 (Tabachnick et al., 2007). Moreover, the Mahalanobis distance test confirmed the absence of multivariate outliers.

The Webpower application was conducted to examine the univariate and multivariate normality. In this case, the skewness value of all continuous variables extended from 0.000 to 0.000, which lies between −3 and +3 (Decarlo, 1997) (see Table 1), indicating no univariate normality issue. Additionally, Mardia’s skewness (β = 0.895, ρ < 0.05) and kurtosis values (β = 31.306, ρ < 0.05) (see Table 1) also unraveled the data freer of multivariate normality.

**TABLE 1:** Mean, standard deviation, data normality.

Construct	Mean	Std. Deviation		Skewness		Kurtosis
Managerial Ties	2.854	0.789		0.056		0.535
Business Ties	2.796	0.955		0.097		−0.120
Political Ties	2.898	0.876		0.061		0.106
Frugal Innovation	2.832	0.737		−0.003		0.695
Sustainable Leadership	2.874	0.839		−0.010		0.264
** Mardia’s multivariate skewness and kurtosis**						

	**b**		**Z**		***p*-value**	

Skewness	0.895		72.049		0.002	
Kurtosis	31.306		−4.850		0.000	

#### Demographic Analysis

The demographics analysis revealed predominantly male participants, *n* = 204, 56.19%. Most participants (*n* = 177, 48.76%) fell in the age category of 25–35, followed by 36–45 (*n* = 143, 39.39%). Furthermore, 171 out of 363 participants held bachelor’s degrees, followed by those (*n* = 167) with master’s degrees and six with doctorates. Notably, the most significant number of participants (*n* = 189) possessed experience of 5–10 years, while only five participants had more than 20 years of experience. The least participants (*n* = 34) originated from the Sialkot city, while the highest (183 out of 363) belong to Rawalpindi city, followed by the Faisalabad city. In this study, mostly representatives (*n* = 141) work at managerial level followed by those (*n* = 53) who are general managers.

#### Reliability and Validity

The measurement model assessment indicates the reliability and validity of each construct (see Table 2). The indicator loadings of the reflective constructs (business ties, political ties, and sustainable leadership) are more significant than 0.50, which lies between 0.558 and 0.873. Hence, all measurement items have acceptable indicator reliability. Furthermore, the Cronbach’s alpha and composite reliability values are higher than the threshold limit of 0.60 (see Table 2). Thus, all reflective constructs (sustainable leadership, business ties, and political ties) exhibited sufficient internal consistency reliability.

**TABLE 2:** Reliability and validity of variables.

Construct	Items	Loading	Cronbach’s alpha		CR	AVE
**Reflective constructs**						
Sustainable leadership	SL1	0.742	0.943		0.949	0.556
	SL2	0.695				
	SL3	0.778				
	SL4	0.652				
	SL5	0.783				
	SL6	0.717				
	SL7	0.812				
	SL8	0.831				
	SL9	0.612				
	SL10	0.820				
	SL11	0.826				
	SL12	0.814				
	SL13	0.558				
	SL14	0.674				
	SL15	0.799				
Business ties (BoT)	BT1	0.808	0.823		0.840	0.637
	BT2	0.747				
	BT3	0.837				
Political ties (PoT)	PT1	0.747	0.803		0.859	0.605
	PT2	0.683				
	PT3	0.873				
	PT4	0.797				

**Formative construct**	**Item**	**Outer Weigh**		**T-Statistic**		**VIF**

Managerial ties	BoT	0.734		11.167		1.337
	PoT	0.401		5.107		1.337
Frugal innovation	FI1	0.115		2.611		1.774
	FI2	0.092		2.330		1.954
	FI3	0.128		3.971		1.265
	FI4	0.543		12.608		2.101
	FI5	0.407		7.560		2.016

The convergent validity of reflective constructs is reportedly based on factor loadings and AVE. All items of reflective variables presented factor loadings greater than 0.60 and AVE values higher than 0.50. In this study, the AVE of SL, BT, and PT are 0.556, 0.637, and 0.605, respectively (see Table 2). Therefore, factor loadings and AVE values confirm the acceptable convergent validity of the reflective construct.

The discriminant validity of variables was tested based on the Fornell-Larcker criterion and HTMT Ratio. None of the corresponding correlation values of any variable is more significant than the square root of its AVE value; hence, all variables exhibited acceptable discriminant validity (see, Table 3). The HTMT ratio confirmed the acceptable discriminant validity as all values were below 0.90 (see, Table 4). However, this study did not assess the reliability of managerial ties and frugal innovation based on Petter et al. (2007) recommendations. The validity was evaluated based on indicator weight, T-values, and VIF using a two-stage approach. The indicator weights of managerial ties and frugal innovation are significant as their VIF values are below 3.3 (see, Table 2), proving acceptable validity.

**TABLE 3:** Correlation values and Fornell-Larcker criterion.

	1	2	3	4	5
BT	***0.798***				
FI	0.174				
MT	0.709	0.264			
PT	0.182	0.100	0.454	***0.778***	
SL	0.133	0.783	0.211	0.080	***0.746***

*Bold and italic values refer to the square root of their corresponding AVE values.*

**TABLE 4:** Heterotrait-Monotrait Ratio (HTMT).

	1	2	3
BT			
PT	0.629		
SL	0.215	0.386	

#### Common Method Bias

The insensitive role of Harman’s factor test and correlation matrix procedure was conducted (Bagozzi et al., 1991; Podsakoff et al., 2012) to investigate the presence of common method bias. The first factor only accounts for 40.57% of the total variance; thus, Harman’s factor test suggested no issue in common method bias. Meanwhile, the correlation matrix procedure negates the presence of common method bias as correlation values are less than 0.90. By using AMOS, a comparison of fit was done between the one-factor model and the measurement model. The results revealed the fit of measurement model (*CFI*0.9660.95;*GFI*0.9530.95;*SRMR*0.0710.08; *RMSEA*0.0840.08) as compared to the one-factor model (*CFI*0.9460.95;*GFI*0.9490.95;*SRMR*0.0790.08; *RMSEA*0.0770.08), confirming the unlikelihood of the common method bias.

#### Descriptive Analysis

The presence of a variable is low if the mean value is equal to or below 2.99, medium in the range of 3.00-3.99, and high if it is more significant than 4.00 (Sekaran and Bougie, 2016). Descriptive analysis revealed that the mean values of all variables ranged between 2.796 and 2.898 (see Table 1), in which the mean values lie below 2.99. Hence, all variables presented a low presence in SMEs of Pakistan, indicating that organizations are putting diminished efforts in this regard. Contrastingly, a study examined sustainable leadership in the HEIs of Pakistan and China, revealing a moderate level Iqbal and Piwowar-Sulej, 2022).

Similarly, frugal innovation was indicated at a medium level in large manufacturing firms (Iqbal et al., 2021). Meanwhile, Wu and Peng (2020) concluded a high presence of political and business ties in China’s SMEs. Thus, previous studies and current empirical evidence reinforce the significance of business ties, managerial ties and sustainable leadership. Ultimately, these findings potentially spur such practices among Pakistan’s SMEs.

#### Direct Hypothesis Testing

The results of the PLS-SEM analyses (see Table 3) indicate that managerial ties significantly influence frugal innovation (β0.507,ρ0.005) among Pakistan’s SMEs, supporting hypothesis H1. Furthermore, empirical evidence supported the significant impact of business ties on frugal innovation (β0.382,ρ0.005). Nevertheless, political ties do not significantly affect frugal innovation (β0.042,ρ0.5580.005) in these SMEs. Thus, hypothesis H1a is accepted, and henceforth, rejecting H1b.

#### Moderating Effect

Hypothesis 2 predicts the positive moderating impact of sustainable leadership on the managerial ties and frugal innovation link. Meanwhile, hypotheses 2a and 2b anticipate that sustainable leadership positively moderates the relationship of business ties and political ties with frugal innovation. Current data analysis found that the coefficient values of managerial ties multiplied by sustainable leadership are still significantly positive 0.050(see Table 5); thus, supporting hypothesis H2. Table 4 and Graph-1 shows the interaction plot indicating the strengthening impact of managerial ties on frugal innovation in Pakistani SMEs. Notably, this phenomenon occurs due to a higher level of sustainable leadership practices.

**TABLE 5:** Direct hypotheses.

Hypotheses	Coefficient	STDEV	*T*-Value	*P*-Values	LLCI	ULCI
Managerial Ties – > Frugal Innovation	0.385	0.079	4.138	0.000	0.173	0.487
Business Ties – > Frugal Innovation	0.330	0.0798	4.138	0.000	0.173	0.487
Political Ties – > Frugal Innovation	0.104	0.108	0.968	**0.333**	0.107	0.317
Managerial Ties × Sustainable Leadership – > Frugal Innovation	0.119	0.041	2.883	0.004	0.037	0.200
Business Ties × Sustainable Leadership – > Frugal Innovation	0.104	0.025	4.122	0.000	0.054	0.154
Political Ties × Sustainable Leadership – > Frugal Innovation	0.031	0.035	0.890	**0.374**	−0.037	0.099

*Values greater than 0.05 are indicated in bold.*

Current empirical evidence shows that interaction term (business ties into sustainable leadership) significantly influences frugal innovation (β0.060,ρ0.0090.050) (see Table 5). The present study confirms the moderating role of sustainable leadership on the business ties-frugal innovation relationship, supporting hypothesis H2a. With increasing values of sustainable leadership practices, impacts of business ties on frugal innovation gets stronger (see, Table 6). Similarly, the interaction plot (see Graph. II) suggests that sustainable leadership in Pakistan’s SMEs intensifies the effect of business ties on frugal innovation.


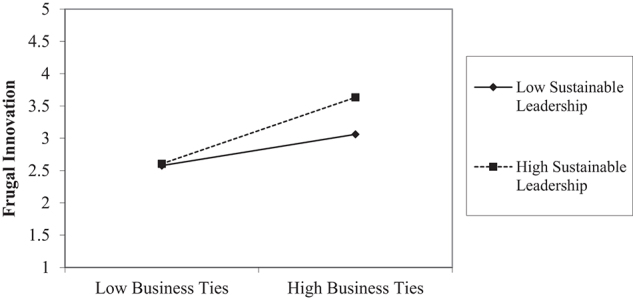


Graph-1: Moderating effect of Managerial Ties.


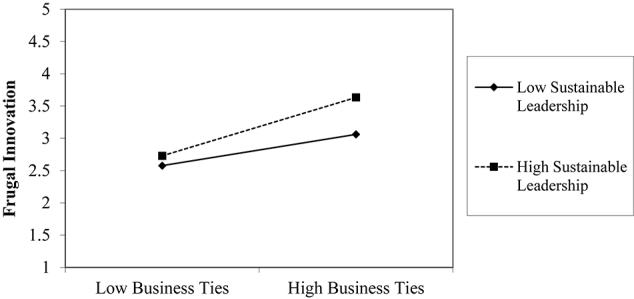


Graph-II: Moderating effect of Business Ties.

**TABLE 6:** Conditional effects of the independent variables at values of the moderator(s).

Sustainable leadership	Effect	se	*t*	*p*	LLCI	ULCI
**Independent variable: Business ties**						
Low	0.043	0.01900	2.263	0.006	0.080	0.043
Moderate	0.067	0.02300	2.913	0.022	0.112	0.067
High	0.100	0.03300	3.030	0.035	0.165	0.100
** Independent variable: Managerial ties**						
Low	0.051	0.02400	2.125	0.004	0.098	0.051
Moderate	0.067	0.02900	2.310	0.010	0.124	0.067
High	0.123	0.04700	2.617	0.031	0.215	0.123

The coefficient value of political ties multiplied by sustainable leadership is approximately zero and non-significant (β0.019,ρ0.050) (see Table 5). Therefore, sustainable leadership does not significantly moderate the relationship of political ties with frugal innovation; henceforth, rejecting hypothesis 2b.

## Discussion

This study attempted to answer a common question in the innovation management field: how to spur frugal innovation in developing countries, and what are favorable conditions for its development? Based on social networking theory and situational leadership theory, a research model was proposed and tested. Specifically, the proposed model is utilized to examine the impact of managerial ties on frugal innovation in the presence of sustainable leadership. The current empirical findings concluded that the relationship between managerial and business ties with frugal innovation strengthens in a higher level of sustainable leadership. Accordingly, this study has provided empirical support favoring four hypotheses, where the results are elaborated below.

The social networking theory was employed to relate managerial ties and their two dimensions, business and political ties, with frugal innovation. The claimed positive relationship between these constructs validated hypotheses H1 and H1a. Hence, this confirmation of propositions H1 and H1a encourage practitioners, top management, ad policymakers to develop and foster their networking. This idea can be further enhanced by strongly bonding with business partners, competitors, customers and suppliers. Past studies provided similar results to current empirical evidence based on hypotheses H1 and H1a (Bell, 2005; Guo et al., 2013; Naqshbandi, 2016).

A study among top managers in UAE concluded that managerial ties significantly influence inbound and outbound innovation (Naqshbandi, 2016). Guo et al. (2013) concluded with a significant impact of managerial ties on the business model innovation. Another study found that centrality in the managerial ties network significantly affects firm innovation (Bell, 2005). Previous authors confirmed this effect of business ties on various innovation factors, i.e., the exploitative (Wu and Peng, 2020), explorative (Su and Yang, 2018; Wu and Peng, 2020), and product (Sami et al., 2019). Other innovation facets include firm (Shu et al., 2012) and social entrepreneurship intentions (Latif and Ali, 2021).

Contrary to current empirical findings, an inverted U-shaped impact of business ties were reported on product innovation (Gao et al., 2017) and radical innovation (Chen et al., 2014). Meanwhile, a non-significant effect of business ties on innovation was reported in foreign direct investment community groups (Gao et al., 2008). Past studies also linked numerous negative consequences concerning business ties, such as time consumption, reciprocals obligations, and maintenance costs (Wang and Chung, 2013; Chung et al., 2016). In this study, the positive impact of political ties was negated on frugal innovation in Pakistan’s SMEs, thus, rejecting hypothesis H1b. Similar to the present empirical evidence, there is no impact of political ties on product innovation in Iran (Sami et al., 2019). Moreover, political ties do not influence exploratory innovation (Su and Yang, 2018). Finally, a study conducted among SMEs in Pakistan and Bangladesh revealed a significant negative effect of political ties on social entrepreneurial intentions (Latif and Ali, 2021).

Contrary to current findings, political ties emerged as a strong predictor of radical (Chen et al., 2014; Shen et al., 2019) and exploitative innovation (Wu and Peng, 2020) among China’s SMEs and manufacturing firms. Hence, the role of political ties has declined in transitional China (Shu et al., 2012). Meanwhile, a U-shaped impact of political ties was indicated on product innovation (Gao et al., 2017). Firms potentially face government involvement in their internal matters, such as employment, conflict of interest, and information blockage in the presence of political ties (Wang and Chung, 2013).

The present study demonstrated the moderating role of sustainable leadership on several constructs based on situational leadership theory. The constructs include managerial, business, and political ties with frugal innovation among Pakistan SMEs. However, current findings exclusively confirm the moderating role of sustainable leadership on the “managerial ties-frugal innovation” and “business ties-frugal innovation” relationship. This result suggests the acceptance of hypotheses H2 and H2a, aligning with the present empirical by Wu and Peng (2020). The study concluded a positive significant conditional effect of empowering leadership on the correlation of business ties with exploratory and exploitative innovation.

The effect of political ties on the exploratory innovation reduces in the presence of a higher level of empowering leadership. However, this effect is insignificant in the exploitative innovation in China’s SMEs (Wu and Peng, 2020). Previous studies confirmed the positive moderating role of numerous variables such as market forces (Chen et al., 2014) and gender, which is more significant for males (Latif and Ali, 2021). Others include innovation orientation and absorptive capacity (Su and Yang, 2018) on the relationship of business ties with radical innovation, exploratory innovation, and social entrepreneurial intentions.

Despite receiving prominent attention, there is limited research on the moderators’ role in the political ties and innovation link (Chen et al., 2014; Gao et al., 2017; Su and Yang, 2018; Latif and Ali, 2021). A study reported a moderating effect of various constructs in China’s firms, i.e., demand uncertainty, technological turbulence, and competitive intensity. Specifically, this effect is subjected to the political ties and radical innovation link (Chen et al., 2014). Meanwhile, another study in China suggested that innovation orientation and absorptive capacity negatively moderate the non-significant political ties and exploratory innovation link (Su and Yang, 2018). A sample study from Pakistan and Bangladesh revealed a negative impact of political ties on social entrepreneurial intentions (Latif and Ali, 2021). However, this impact is enhanced and becomes more robust in the presence of females. Other studies found a moderating impact of the micro-institutional environment on the curvilinear relationship between business and political ties. This link is assessed with product innovation in China’s industrial sector (Gao et al., 2017).

### Implications

The present study offers both theoretical and practical implications. From the perspective of theoretical implications, first, the current research significantly contributes to the literature on managerial ties and frugal innovation as it provided empirical evidence about their relationship. Second, the present research is first in its nature to investigate the integrated relationship between business ties, political ties, sustainable leadership and frugal innovation. Third, this study has enriched the literature on social networking theory by examining the impact of managerial ties, business ties and political ties on frugal innovation. Fourth, the current research also enhanced the literature on situational leadership theory by assessing the conditional effect of sustainable leadership on the relationship of managerial ties, business ties and political ties with frugal innovation.

Based on current empirical findings, three managerial implications are suggested, which provide guidance for SMEs on employing managerial ties to stimulate frugal innovation. Firstly, this study claims that business ties positively impact frugal innovation, though they are actually impacted by political ties. This phenomenon indicates that SMEs in Pakistan can obtain the necessary resources from business partners to innovate frugally. Hence, Pakistan’s SMEs should cultivate and maintain business ties with other firms, customers, and suppliers, expanding channels and product sales.

In Pakistan, political ties are not perceived as an adequate resource to promote frugal innovation. This relationship might be due to the dysfunctional market, a low influx of government funds, insufficient automation, and poor communication channels (Shah, 2018; Iqbal et al., 2021b). The low influx of funds is only enough for their regular business operations. Furthermore, its government offices and officials neglect their focus on the SMEs’ needs and abandon laws and regulations that benefit these enterprises. Public banks in Pakistan hesitate to approve loans to SMEs because of insufficient documentation and repayment facilities.

Notably, SMEs face difficulties comprehending the rules and regulations introduced by government offices, which are time-consuming because of the excessive paperwork (Iqbal et al., 2020d). These factors might hinder the positive impact of political ties on frugal innovation (Ünlü and Alshebami, 2022). Thus, policymakers, government officials, and SME representatives are advised to resolve these issues and work together to align their strategies, developing novel approaches to spur frugal innovation. This idea will consequently help SMEs work in close liaison with political offices to serve their community under the national development goals.

Secondly, sustainable leadership was reported to positively moderate the relationship of managerial ties and business ties with frugal innovation. The findings claim the effectiveness of sustainable leadership, encouraging SMEs to focus on promoting this concept. Generally, SMEs are not able to effectively employ excellent managerial ties without underestimating the crucial role of sustainable leadership. However, most SMEs in developing countries are not cognisant of this idea, which was initially introduced in developed countries, i.e., Australia. This idea presents a vital indicator of sustainable leadership than other management styles deemed less effective in coping with sustainable challenges. In short, SMEs can use this study as a benchmark to improve their resources and competitiveness to flourish and innovate. Simultaneously, they must observe and adapt to the market needs in a highly dysfunctional competition.

## Conclusion

The objective of the present study was to investigate the direct relationship of managerial ties and its two dimensions- business ties and political ties, with frugal innovation and moderating impact of sustainable leadership on their proposed relationship in the developing countries. In this study, cross-sectional data is collected from manufacturing firms in Pakistan. The empirical findings confirmed the positive impact of managerial ties and business ties on frugal innovation but did not support the direct relationship between political networking with frugal innovation. Moreover, current research concluded with the positive moderating effect of sustainable leadership on the relationship of managerial networking and business ties with frugal innovation. Yet, sustainable leadership do not moderate the political ties-frugal innovation relationship in the developing economies such as Pakistan.

The present study presented various limitations that require vigilant interpretations of the empirical findings, which provide opportunities for future research. Firstly, generalization issues were present as the study was conducted among SMEs in Pakistan. The effectiveness of managerial ties is interlinked with the institutional, organizational, and strategic context unique to an industry and region (Chung et al., 2016). Hence, future studies are encouraged to be conducted in other regions to cope with the generalization issues. Secondly, the moderating role of sustainable leadership was exclusively investigated on the managerial ties-frugal innovation relationship. However, the study did not consider other possible factors such as innovation orientation (Su and Yang, 2018), market turbulence, and competitive intensity (Chen et al., 2014). Future researchers should consider the dark side of the business and political ties concerning frugal innovation in the presence of sustainable leadership as a conditional factor.

Thirdly, this study has only collected data from SMEs in prominent cities, namely Lahore, Faisalabad, Gujranwala, Rawalpindi, and Sialkot. These territories come under the jurisdiction of Punjab province, Pakistan. Future studies are therefore suggested to extend their sample size by collecting data from varying provinces of Pakistan, increasing the representation of the findings. The final limitation includes the lack of analysis on managerial ties-frugal innovation relationship mechanism. According to the author’s knowledge, only Naqshbandi (2016) reported the role of absorptive capacity as a mediator, specifically in the managerial ties-open innovation relationship. Therefore, future endeavors are advised to explore other possible mechanisms for the relationship of managerial ties with frugal innovation.

## Data Availability Statement

The original contributions presented in this study are included in the article/supplementary material, further inquiries can be directed to the corresponding author/s.

## Author Contributions

Both authors listed have made a substantial, direct, and intellectual contribution to the work, and approved it for publication.

## Conflict of Interest

The authors declare that the research was conducted in the absence of any commercial or financial relationships that could be construed as a potential conflict of interest. The reviewer ZK declared shared affiliation with the author WX to the handling Editor at the time of review.

## Publisher’s Note

All claims expressed in this article are solely those of the authors and do not necessarily represent those of their affiliated organizations, or those of the publisher, the editors and the reviewers. Any product that may be evaluated in this article, or claim that may be made by its manufacturer, is not guaranteed or endorsed by the publisher.

## Appendix

**Appendix-A | Measurement items.**

Managerial ties

***Business ties***

The extent to which top managers at your organization utilize personal ties, networks, and connections with

∙ with top managers at buyers firms

∙ with top managers at supplier firms

∙ top managers at competitors firms

***Political ties***

The extent to which top managers at your organization utilize personal ties, networks, and connections with

∙ political leaders in various levels of the government.

∙ officials in industrial bureaus.

∙ officials in regulatory organizations such as tax bureaus.

∙ Officials in supporting organizations such as state banks

**Frugal Innovation**

∙ Our organization develop low-cost product/services.

∙ It is easy-to-use our organizational product/service.

∙ Our organization uses resources thriftily.

∙ Our organization offers sustainable product/service.

∙ Our organization introduces good-enough quality products/services.

**Sustainable leadership**

∙ Leaders in your organization act in a sustainable socially responsible manner.

∙ Leaders in your organization act in a sustainable environmentally responsible manner.

∙ Leaders in your organization act in a sustainable ethically responsible manner.

∙ Leaders in your organization make decisions while considering the entire organization.

∙ Leaders in your organization officially recognize when a mistake is made that affects sustainability.

∙ Leaders in your organization are willing to correct mistakes that affect sustainability.

∙ Leaders in your organization attempt to use unique innovative methods to resolve sustainability issues.

∙ Leaders in your organization attempt to create wealth through sustainable efforts.

∙ Leaders in your organization put purpose before profit.

∙ Leaders in your organization balance sustainable social responsibility with profits.

∙ Leaders in your organization demonstrate sustainability by persevering through all types of change.

∙ Leaders in your organization are concerned how sustainability affects employees.

∙ Leaders in your organization communicate sustainability decisions to all involved.

Leaders in your organization attempt to build a culture of sustainability through its communication efforts.
